# Relevance of Amorphous and Amyloid-Like Aggregates of the p53 Core Domain to Loss of its DNA-Binding Activity

**DOI:** 10.3389/fmolb.2022.869851

**Published:** 2022-04-26

**Authors:** Emi Hibino, Takeshi Tenno, Hidekazu Hiroaki

**Affiliations:** ^1^ Laboratory of Structural Molecular Pharmacology, Graduate School of Pharmaceutical Sciences, Nagoya University, Nagoya, Japan; ^2^ BeCellBar LLC., Nagoya University, Nagoya, Japan

**Keywords:** thioflavin-T, tumor suppressor p53, amyloid aggregation, DNA-binding domain, p53 core domain, amorphous aggregation

## Abstract

The anti-oncogenic protein p53 is a transcription factor that prevents tumorigenesis by inducing gene repair proteins or apoptosis under DNA damage. Since the DNA-binding domain of p53 (p53C) is aggregation-prone, the anti-oncogenic function of p53 is often lost in cancer cells. This tendency is rather severe in some tumor-related p53 mutants, such as R175H. In this study, we examined the effect of salts, including KCl and sugars, on the aggregation of p53C by monitoring two distinct aggregates: amorphous-like and amyloid-like. The amorphous aggregates are detectable with 8-(phenylamino)-1-naphthalenesulfonic acid (ANS) fluorescence, whereas the amyloid aggregates are sensitive to thioflavin-T (ThT) fluorescence. We found that KCl inhibited the formation of amorphous aggregates but promoted the formation of amyloid aggregates in a p53C R175H mutant. The salts exhibited different effects against the wild-type and R175H mutants of p53C. However, the ratio of ANS/ThT fluorescence for the wild-type and R175H mutant remained constant. KCl also suppressed the structural transition and loss of the DNA-binding function of p53C. These observations indicate the existence of multiple steps of p53C aggregation, probably coupled with the dissociation of Zn. Notably, amorphous aggregates and amyloid aggregates have distinct properties that could be discriminated by various small additives upon aggregation.

## Introduction

The oncogenic suppressor protein p53 is a transcription factor that induces the expression of genes involved in the DNA repair process when DNA damage occurs, or that induces apoptosis when DNA damage is severe. Thus, p53 is called “the guardian of the genome” because it prevents cells from tumorigenesis (Lane, 1992). Reduced p53 function is shown to promote cancer, and half of all cancers show mutations in p53.

p53 harbors three functional/structural domains—the DNA-binding domain or core domain (p53C), the transcriptional activation domain, and the tetramerization domain. Particularly common mutations in cancer are called hotspot mutations, which are frequently observed in the p53C (Olivier et al., 2002; Bouaoun et al., 2016; Wang et al., 2016). p53 with hotspot mutations is further classified into two classes: contact mutants and structural mutants (Wong et al., 1999; Bullock et al., 2000; Ishimaru et al., 2003b; Hibino and Hiroaki., 2022). Some severe p53 mutants are no longer able to bind to their native target DNA sequence (Kim and Lozano, 2018).

p53C is aggregation-prone (Ishimaru et al., 2003a) and its aggregative tendency increases with hotspot mutations (Ishimaru et al., 2003a; Pedrote et al., 2018). At least two types of p53C aggregates—amorphous-like aggregates and amyloid-like aggregates—have been reported. However, the detailed mechanisms of the aggregation processes, including whether the amorphous and amyloid aggregates form simultaneously or distinctly, remain unclear (Ishimaru et al., 2004; Ghosh et al., 2017; Yang et al., 2021). Moreover, many hotspot mutations seem to represent dominant negative phenotypes, such that the functional wild-type p53 proteins are entrapped in the aggregates formed by the mutant molecules, and these aggregates even propagate to gain certain toxicity (Ano Bom et al., 2012; Wang and Fersht, 2015; Garg et al., 2020). Accordingly, it is assumed that the Zn coordinating to p53 can contribute to the structural stabilization of p53, whereas the removal of Zn destabilizes the overall structure (Duan and Nilsson, 2006). The dissociation of Zn triggers overall aggregation (Butler and Loh, 2003). A process of the structural transition between Zn-binding form and unstructured form without Zn of p53 can be monitored by the change in fluorescence of tryptophan and tyrosine residues in the p53 polypeptide (Rangel et al., 2019).

Accordingly, a therapeutic strategy of inhibiting p53 aggregation (Shigemitsu and Hiroaki, 2018) has been challenged by many research groups, and several candidates have been reported, including ReAC peptide, resveratrol, PRIMA-I, pk9318, and so on (Soragni et al., 2016; da Costa et al., 2018; Bauer et al., 2019; Rangel et al., 2019). We also hypothesize that some sugars and/or osmolytes can inhibit p53 aggregation by stabilizing the monomeric p53 molecules, similar to the osmolyte inhibition of Aβ (1–42) fibril formation, which we previously reported (Iwaya et al., 2020).

Thus, we further attempt to describe a mechanism for suppressing p53 aggregation to maintain its function, because p53 is aggregation-prone even in the wild-type, and the p53 aggregates can propagate and inactivate healthy p53 molecules, which results in a dominant negative phenotype (Iwashita et al., 2018; Shiraki et al., 2020).

In this study, we focused on several additives (salts and osmolytes), which are important for cell maintenance. We have previously studied the effects of osmolytes and phenolic compounds on amyloid aggregation of Aβ (Iwaya et al., 2020). Thus, we decided to investigate the effect of small molecules that control p53 aggregation. The aggregation tendencies of p53 in the presence and absence of various additives, including potassium ion and glucose, were investigated for wild-type p53C (p53C-wt) and p53C R175H (p53C-R175H). The R175H mutation is one of the hotspot mutations classified as a structural mutation and has been reported to promote aggregation (Butler and Loh, 2003). We employed two different fluorescent dyes simultaneously: 8-(phenylamino)-1-naphthalenesulfonic acid (ANS), which can detect amorphous aggregates, and Thioflavin T (ThT), which can detect amyloid aggregates (Khan et al., 2014).

We succeeded in reproducing the result that the amorphous aggregates could be suppressed in a concentration-dependent manner by KCl, as reported previously (Kovachev et al., 2017). However, we found that KCl did not affect the formation of the amyloid aggregates of the p53C-wt, and even accelerated the formation of amyloid of p53C-R175H. All salts examined in this study increased the ratio of ThT/ANS fluorescence, whereas glucose and trehalose did not affect the ThT/ANS ratio. Moreover, under conditions of promoted amyloid aggregation, the ability of DNA binding was retained. The relationship between Zn binding and DNA binding activity upon p53C aggregation is further discussed.

## Materials and Methods

### Protein Expression and Purification

The expression vector for the recombinant GST-tagged form of the DNA-binding domain of p53 (residues 94–312) was constructed. To prepare a plasmid for p53C-wt expression, the genomic sequence encoding the corresponding amino acid sequence was amplified with two primers designed from human adult normal kidney cDNA (BioChain Institute, Hayward, CA, United States) and placed into pGEX-6P-3 (Cytiva, Tokyo, Japan) using an In-Fusion HD Cloning Kit (Takara bio, Kusatsu, Japan) according to the manufacturer’s protocol. To prepare a plasmid for p53C-R175H expression, two complementary oligonucleotides with the mutated sequence were used as primers to introduce a mutation that replaced the amino acid encoding Arg175 with His. The protein was prepared by expression in *Escherichia coli* BL21 (DE3), and the cells were cultured in LB medium. Isopropyl-β-D-thiogalactopyranoside was added at an OD_600_ of approximately 0.6 to a final concentration of 0.2 mM, and the cells were incubated at 20°C overnight. The harvested cells were resuspended in lysis buffer (20 mM phosphate buffer, pH 6.0, 300 mM NaCl) and disrupted by sonication. The supernatant was applied to a DEAE-Sepharose (Cytiva) column and affinity purified using Glutathione Sepharose 4 Fast Flow (Cytiva) chromatography. The GST tag was removed by HRV 3C protease on beads. The protein was subsequently purified by size exclusion chromatography using a HiLoad 26/60 Superdex 75 pg (Cytiva) in 20 mM phosphate buffer, pH 6.0, 140 mM NaCl, and 1 mM dithiothreitol (DTT). After purification, the sample was concentrated on an ultrafiltration membrane and stored at −80°C. For use, the samples were dialyzed in an assay buffer of 20 mM Tris-HCl and 1 mM DTT at pH 7.3. After dialysis, the supernatant was separated by centrifugation at 15,000 ×g at 4°C for 2 min and placed on ice until just before use.

### Fluorescence Measurements on a Fluorescence Spectrophotometer

The aggregation of p53C was monitored by fluorescence enhancement upon both ANS (Sigma-Aldrich; Merck KGaA) and ThT (Sigma-Aldrich) binding (Yoshimura et al., 2012; Adachi et al., 2015; So and Yoshimura, 2020). ANS and ThT were added simultaneously to contain 10 and 20 μM, respectively, and the concentration of the p53 protein was 4 μM. The measurement samples were prepared at room temperature within 2 min, aliquoted into a quartz cell pre-warmed with the cell holder to 37°C in an F-7000 fluorescence spectrophotometer (Hitachi High-Tech Co., Tokyo, Japan), and measured immediately using the FL Solutions program of the instrument. The fluorescence intensity was measured every 2 min for 1 h. The fluorescence wavelength was set to 485 nm, the excitation spectrum was scanned, and the values at 375 and 445 nm were extracted as the fluorescence of ANS and ThT, respectively.

### Fluorescence Measurements on Microplate Reader

The ANS/ThT values after 60 min of aggregation were measured with an EnSpire Multimode Plate Reader (PerkinElmer Inc., Waltham, MA, United States). The p53C solution at a concentration of 4 μM with 10 μM ANS and 20 μM ThT was prepared on a black 96-well plate (OptiPlate-96 F; PerkinElmer Inc.) on ice and measured before incubation to use as the blank. After incubation at 37°C for 1 h, the reaction was stopped by placing the plate on ice. The excitation wavelength was set to 375 and 445 nm for ANS and ThT, respectively, and the detection wavelength was set to 485 nm for both fluorophores. Each sample was measured three times, and the average of the measurements was used as a single datum. The samples were *n* = 3, and the mean and standard deviations were calculated.

### Fluorescence Microscopy

p53C solution at a concentration of 4 μM was incubated on a transparent 96-well microplate (AGC Techno Glass Co., Ltd., Shizuoka, Japan) for 2 h at 37°C. Both ThT and ANS were added to a concentration of 40 μM, and observation was performed. The fluorescence images were obtained at ×10 magnification using fluorescence microscopy (IX-71; Olympus, Tokyo, Japan).

### Nanoparticle Tracking Analysis

After aggregation, all samples were diluted 100 times in a buffer of 20 mM Tris-HCl and 1 mM DTT at pH7.3 to a final volume of 1 ml. Measurements on Nanosight NS300 (Malvern, United Kingdom) were executed according to the manufacturer’s software manual. The measurements were performed at 25°C. The sample solution was collected in a 1 ml disposable syringe and measured while pumping with a syringe pump. The NTA 2.3 software was used for video capture and data analysis. The data of the five measurements were averaged, and the standard deviation was calculated.

### DNA Binding Assay

The DNA to bind p53 was prepared by introducing AGG​CAT​GCC​TAG​GCA​TGC​CT (Chen et al., 2010) into pGEX-6P-3 using an In-Fusion HD Cloning Kit. DNA of 100 bp length containing this sequence was amplified using PCR and purified using a Wizard SV Gel and PCR Clean-Up System (Promega, Madison, WI, United States). A solution of 4 μM p53C was incubated at 37°C, and a part was sampled at the appropriate time and placed on ice. When all sampling was completed, 20 ng of purified 100 bp DNA was added, and then the samples were run on a 6% TBE gel. The gel was stained with ethidium bromide and detected on a LED transilluminator (GELmieru; FUJIFILM Wako, Osaka). The band intensities were analyzed using ImageJ software.

### Nuclear Magnetic Resonance

A solution of 15 μM p53C in a buffer of 20 mM phosphate and 10% (v/v) D_2_O at pH 7.3 was incubated at 37°C and placed on ice. A deuterated glucose (Cambridge Isotope Laboratories, Inc., Andover, MA, United States) was used as a glucose for NMR measurements. One-dimensional ^1^H-NMR was measured at 15°C on a 900 MHz NMR spectrometer (Avance III; Bruker, Karlsruhe, Germany) equipped with a cryogenic triple-resonance probe. All NMR data were obtained using Topspin software and analyzed using NMRPipe software packages.

### Tryptophan and Tyrosine Fluorescence Measurements

The fluorescence intensity of tyrosine and tryptophan to detect the structural transition of p53C was monitored on an F-7000 fluorescence spectrophotometer. The concentration of the p53 protein was 4 μM. The measurement samples were prepared at room temperature within 2 min, entered into a quartz cell pre-warmed to 37°C with the cell holder in the fluorescence spectrophotometer, and immediately measured using the FL Solutions program of the instrument. The fluorescence intensity was measured every 2 min for 1 h. The excitation wavelength was set to 280 nm, the fluorescence spectrum was acquired, and then the ratio of the value at 333 nm to the value at 305 nm was calculated.

## Results

### KCl Promotes p53C-R175H Amyloid Aggregation

**FIGURE 1:**
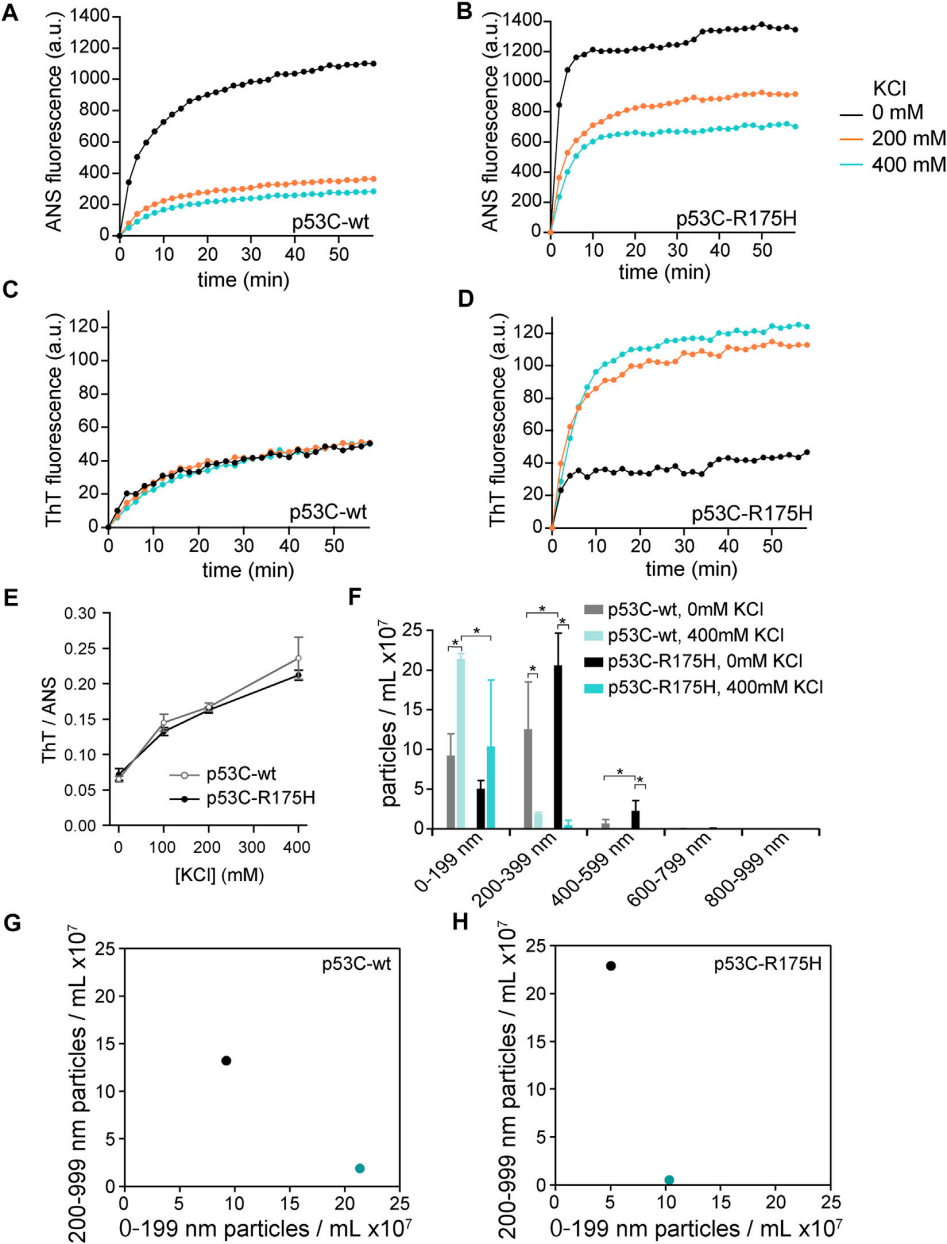
**(A–D)** Effect of KCl on aggregation of p53C-wt and p53C-R175H. p53C solution was aggregated at 37°C in the absence of KCl (black), with 200 mM KCl (orange), or with 400 mM KCl (cyan), and the values were plotted every 2 min. **(A)** ANS fluorescence in p53C-wt; **(B)** ANS fluorescence in p53C-R175H; **(C)** ThT fluorescence in p53C-wt; and **(D)** ThT fluorescence in p53C-R175H. **(E)** The ratio of ThT to ANS of p53C-wt (gray, open circle) and p53C-R175H (black, solid circle) were plotted against the concentration of KCl. **(F)** Results of NTA. The size of the aggregates of p53C-wt and p53C-R175H formed in the absence of KCl or in 400 mM KCl was divided into 200 nm increments. **(G,H)** The number of particles in the size range 0–199 nm is plotted on the horizontal axis and the number of particles in the size range 200–999 nm on the vertical axis for p53C-wt **(G)** and p53C-R175H **(H)**, respectively. The data are identical to those in Panel **(F)**. Black is in the absence of KCl, and cyan is under 400 mM KCl.

The wild-type of p53C (p53C-wt) and R175H mutant of p53C (p53C-R175H), which is a type of hotspot mutation, were reported to aggregate (Butler and Loh, 2003). First, we monitored the aggregation of p53C using two kinds of fluorophores: ANS and ThT. The fluorescence intensity of ANS increases in hydrophobic environments and that of ThT increases by binding to cross-β sheet structures, which is characteristic of amyloid structures (Khan et al., 2015). As a result, the fluorescence intensity of both ANS and ThT increased with time; we were able to reproduce these previous reports (Figures 1A–D). We then examined the effect of the presence of KCl, a type of charged osmolyte in cells, on the aggregation of p53C-wt and p53C-R175H. The increase in the fluorescence intensity of ANS was reduced (Figures 1A,B). Although p53C-wt could not increase or decrease the fluorescence intensity of ThT (Figure 1C), p53C-R175H caused a increase in ThT fluorescence intensity (Figure 1D). There was concern that these fluorescence fluctuations were due to the ionic effect of 400 mM KCl. Thus, we examined the effect of KCl in the presence of either p53C-wt or p53C-R175H. For ANS fluorescence, approximately 180% and 140% fluorescence enhancement were observed against non-aggregated p53C-wt and p53C-R175H, respectively. In contrast, approximately 55% and 45% reduction of ThT fluorescence were observed (Supplementary Figures S2A,B). We showed the normalized fluorescence changes upon aggregation of p53C-wt and p53C-R175H monitored by ANS or ThT (Supplementary Figures S2C,D). Taking all these effects into consideration, we confirmed the tendency of the salt-dependent suppression of the ANS fluorescence and the salt-dependent enhancement of ThT fluorescence. To analyze the change in the ratio of ANS and ThT based on the concentration of KCl, we measured the increased ANS and ThT fluorescence values on the microplate for high-throughput analysis. p53C solution was aggregated for 1 h at 37°C on a 96-well microplate, and we plotted the calculated value of ThT/ANS on the vertical axis and the concentration of KCl on the horizontal axis. The results showed that the value of ThT/ANS increased in a KCl concentration-dependent manner and that the values were similar for p53C-wt and p53C-R175H (Figure 1E).

To clarify the difference in the shape of aggregates with and without 400 mM KCl, we tried to observe the aggregates stained with ANS and ThT by fluorescence microscopy. Aggregates stained with ThT were so few in both p53C-wt and p53C-R175H that they were not visible under fluorescence microscopy at 10-fold magnification, but aggregates stained with ANS were visible. Under conditions without KCl, the aggregates of p53C-R175H were larger than those of p53C-wt (Supplementary Figures S1A,B). In the 400 mM KCl condition, both p53C-wt and p53C-R175H mutants showed much less aggregation (Supplementary Figures S1C,D), which was consistent with the results of the fluorescence spectrophotometer experiments.

Fluorescence microscopy suggested that the morphologies of the p53C-wt and p53C-R175H aggregates differed and were dependent on salt concentrations. Therefore, to analyze the aggregate size in detail, an NTA assay was performed. We incubated the p53C solution at a concentration of 4 μM for 1 h at 37°C and then placed it on ice to stop aggregation. To allow for proper NTA analysis, the samples were diluted 100-fold after aggregation and then assayed (Supplementary Figure S3). To facilitate understanding of the measurement results, the number of particles observed at each 200 nm was summed (Figure 1F). The results showed that in 0 mM KCl, p53C-wt had approximate equal amounts of 0–199 nm and 200–399 nm particles, whereas in 400 mM KCl, the majority of p53C-wt particles were 0–199 nm. The aggregates of p53C-R175H under 0 mM KCl conditions were larger than those of p53C-wt, and there were more particles larger than 200 nm than those under other conditions. The aggregates of p53C-R175H and p53C-wt in 400 mM KCl showed mostly 0–199 nm particles.

For further analysis, the number of particles with a size of 0–199 nm was plotted on the horizontal axis and the number of particles with a size of 200 nm or larger was plotted on the vertical axis (Figures 1G,H). Both p53C-wt and p53C-R175H shifted to the lower right; that is, the particle size became smaller with an increase in salt concentration.

The relationship between the aggregation conditions and the size of the aggregates was consistent with that observed by fluorescence microscopy, although particles with a size of less than 20 μM observed in the fluorescence microscopy could not be detected because they were beyond the detection limit of the NTA assay.

### The Effects of Other Charged Osmolytes and Uncharged Osmolytes

**FIGURE 2:**
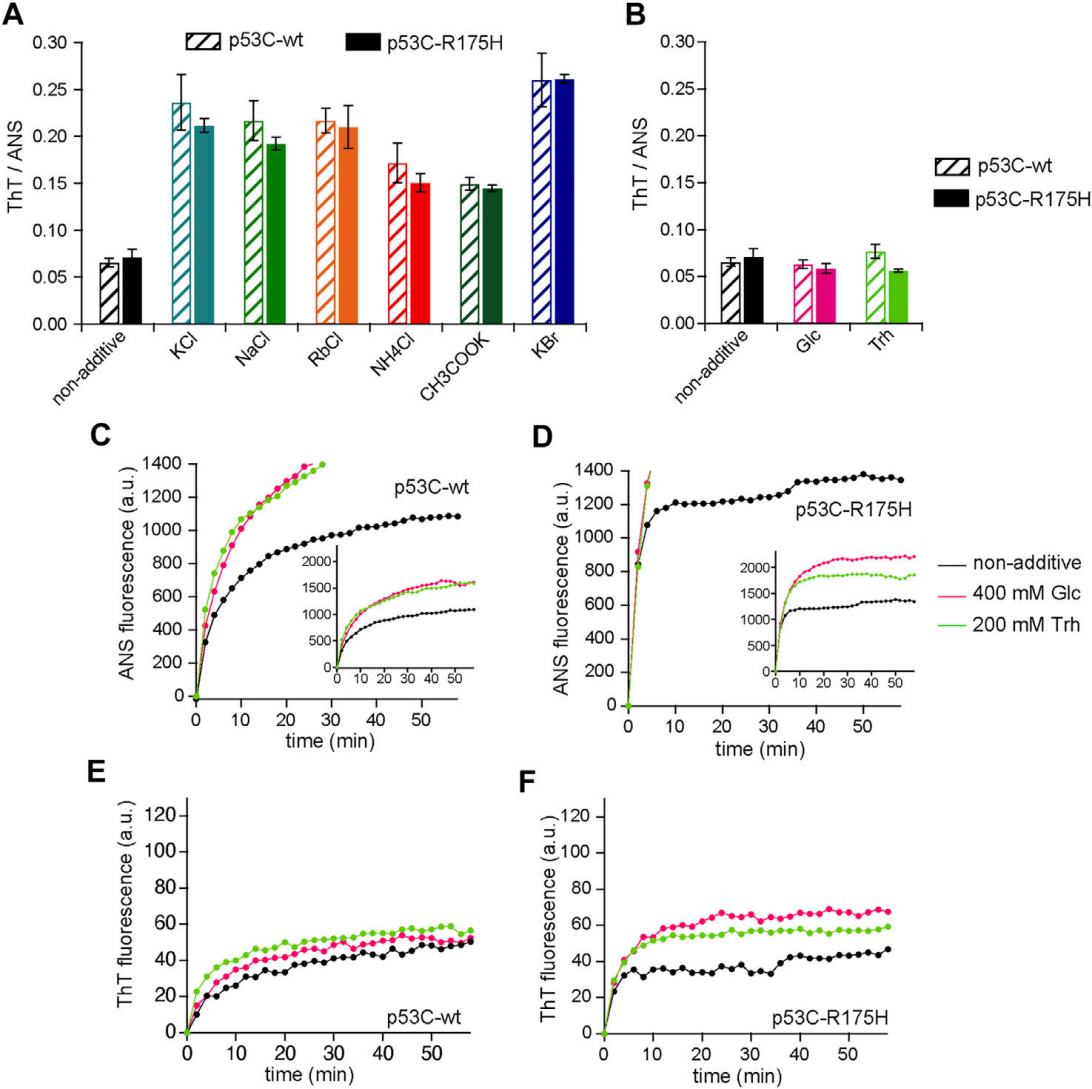
**(A,B)** The ratio of ThT to ANS for various charged small additives **(A)** and uncharged additives **(B)** in p53-wt (shaded bars) and p53-R175H (fill bars). **(C–E)** Effect of glucose and Trh on aggregation of p53C-wt and p53C-R175H. The data for non-additives are identical to those in Figures 1A–D, and have been redrawn for comparison. p53C solution was aggregated at 37°C with 400 mM Glc (magenta) or with 200 mM Trh (light green), and the values were plotted every 2 min. **(C)** ANS fluorescence in p53C-wt; **(D)** ANS fluorescence in p53C-R175H; **(E)** ThT fluorescence in p53C-wt; and **(F)** ThT fluorescence in p53C-R175H. The inset of **(C,D)** is a graph with the vertical axis adjusted to include all the plots.

The aggregation-inhibiting and aggregation-promoting effects of salt follow the Hofmeister series. To clarify the Hofmeister effect on the aggregation of p53C, chloride salts of Na^+^, NH_4_^+^, and Rb^+^ or potassium salts of CH_3_COO^−^ and Br^−^ were added. According to Hofmeister (1888), anions Br^−^, Cl^−^, and CH_3_COO^−^, in that order, and cations NH_4_^+^, Rb^+^, K^+^, and Na^+^, in that order, are more effective in salting out proteins. Upon aggregation in a 96-well microplate at 37°C for 60 min, both p53C-wt and p53C-R175H exhibited a similar fluorescence ratio of ThT/ANS in the presence of each tested salt (Figure 2A; Supplementary Figure S4). Further, the order of the strength of the salting-out effect did not correspond to the order of the increase or decrease in ANS or ThT, suggesting that the inhibition of aggregation was different from that observed in other amyloids due to the salting-in and salting-out effects of ions (Rabbani et al., 2012).

We then examined the effects of glucose (Glc) and trehalose (Trh) on p53 aggregation; these uncharged osmolytes reportedly affect protein aggregation (Iwaya et al., 2020). Since Glc and Trh are monosaccharide and disaccharide, respectively, a concentration of 200 mM was adopted for Trh to match the monosaccharide concentration (Rabbani and Choi, 2018). As in the case of charged osmolytes, we calculated the ratio of ThT/ANS. The results showed that neither Glc nor Trh had any effect on the ratio of ThT/ANS after 60 min of aggregation of p53C (Figure 2B). The changes in the fluorescence intensity of ANS and ThT were measured over time using a fluorophotometer, and we found that the values of both ANS and ThT showed an increasing tendency (Figures 2C–F). Since the fluorescence intensity of ANS and ThT increased in a similar manner, the ratios were close.

### The DNA-Binding Activity of p53 is Retained by Osmolytes

**FIGURE 3:**
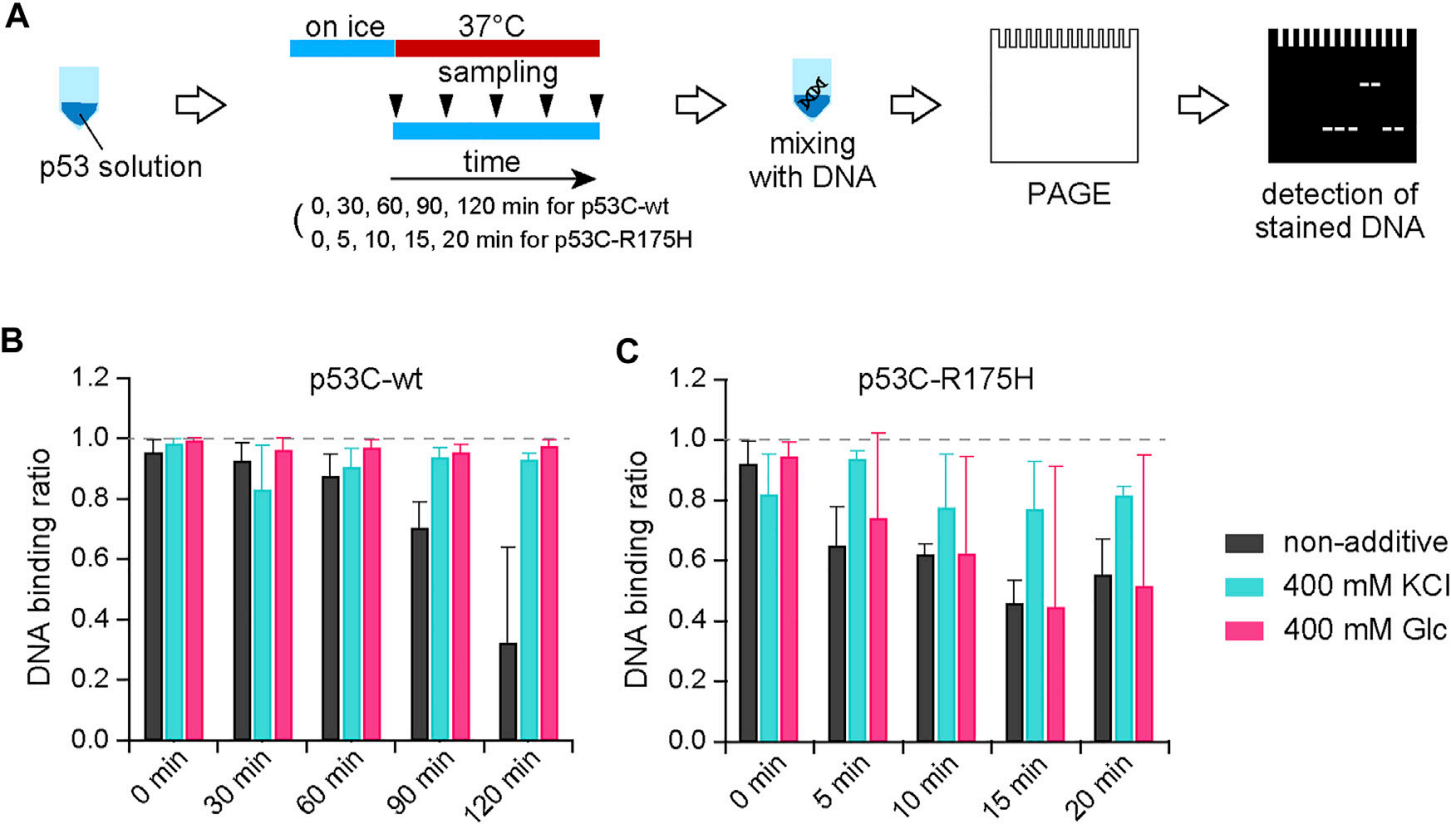
**(A)** Schematic diagram of the experimental procedure for the gel shift assay using p53 and DNA. **(B,C)** The unbound fraction was calculated from the intensity of the band at the location of the DNA without the protein solution and normalized to 1 for bound DNA and 0 for unbound DNA.

Since p53 is a transcription factor, its binding to DNA is essential for its function. To evaluate the effect of KCl on this protein–DNA binding, we performed a gel shift assay. A graphical overview of the experiment is shown in Figure 3A. A p53C solution at a concentration of 4 µM was prepared on ice and incubated at 37°C for 30, 60, 90, and 120 min for the p53C-wt and 5, 10, 15, and 20 min for the p53C-R175H, and then placed on ice again to stop p53C aggregation. The DNA for the assay was a 100-bp sequence that contained a p53C binding site, which was amplified by PCR and purified. The sample was placed on ice and mixed with the 100 bp DNA, and the KCl concentration was adjusted so that the final concentration of KCl was 200 mM, a concentration at which the salt had no effect on p53C-DNA binding (Ishimaru et al., 2009). The DNA was also mixed after aggregation because binding to DNA and RNA stabilizes p53 and inhibits aggregation (Paleček et al., 1997; Ishimaru et al., 2009; Silva et al., 2010; Kovachev et al., 2017). After electrophoresis of the mixture of p53C and DNA on a TBE gel, the gel was stained with ethidium bromide to detect DNA bands, and the protein complex fraction and the unbound DNA fraction were analyzed and normalized by binding rate (Figures 3B,C; Supplementary Figure S5). Unless they were exposed to 37°C, we confirmed that not only p53C-wt but also p53C-R175H retained their DNA-binding capacity. We also found that the DNA-binding activities of p53C-wt and p53C-R175H in samples with no additives were lost because of aggregation. As expected, both p53C-wt and p53C-R175H with KCl retained their DNA-binding ability. The addition of Glc also kept the DNA binding activity of p53C-wt equivalent to that of KCl. However, Glc was not as effective as KCl in maintaining the DNA-binding activity of p53C-R175H. Intriguingly, the DNA-binding activity of p53C-wt was maintained under the conditions of a high ratio of ThT/ANS or increased ThT fluorescence.

### Detection of Structural Transition by One-Dimensional ^1^H-NMR

**FIGURE 4:**
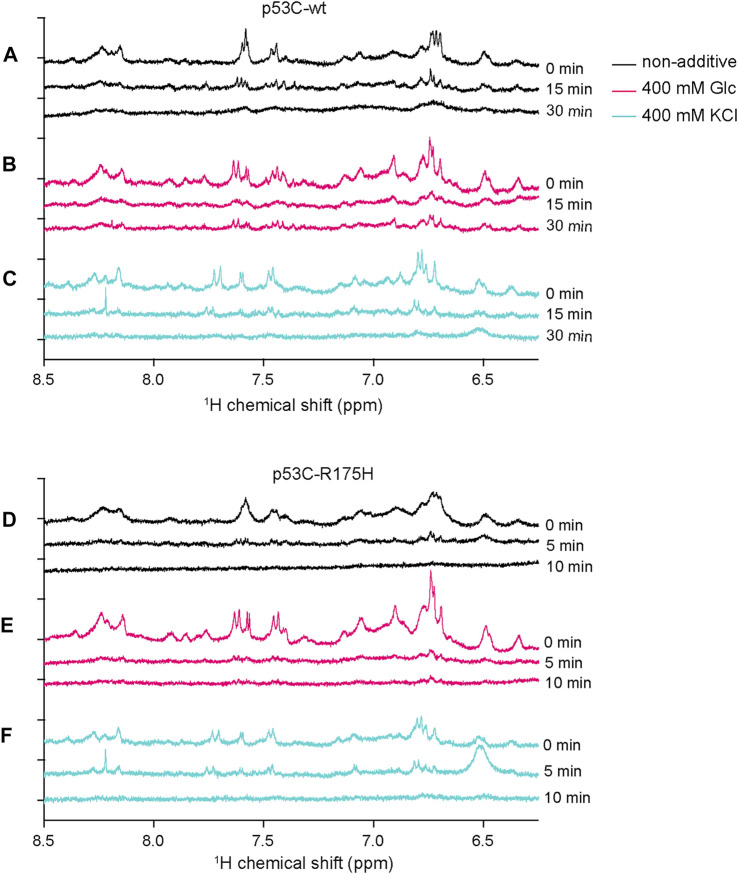
The subtle structural changes associated with the aggregation of the p53C solution were monitored by 1D ^1^H-NMR. **(A–C)** 1D ^1^H-NMR spectra of p53C-wt at 0, 15, and 30 min, without additives (A), with 400 mM deuterated Glc **(B)**, or with 400 mM KCl **(C)**. **(D–F)** 1D ^1^H-NMR spectra of p53C-R175H at 0, 15, and 30 min, without additives **(D)**, with 400 mM deuterated Glc **(E)**, or with 400 mM KCl **(F)**.

To detect the aggregation details of p53C-wt and p53C-R175H with and without KCl or Glc, we obtained one-dimensional ^1^H-NMR spectra. In the NMR experiment, the structure of the monomer should be detectable because the aggregates increase in molecular weight, with the peaks broadening, and should not be detected. Almost all experiments were performed at a protein concentration of 4 μM, but NMR was performed at a concentration of 15 μM to increase the signal-to-noise ratio. To suppress the appearance of peaks other than p53C in the 1D-NMR spectrum, phosphate buffer and deuterated glucose were substituted for Tris-HCl buffer and glucose, respectively, and DTT was not added. Samples were prepared on ice and incubated at 37°C for 15 or 30 min for the p53C-wt, 5 or 10 min for the p53C-R175H, and then placed back on ice to stop aggregation, followed by NMR measurements at 15°C. Note that we checked separately that the NMR spectrum of p53C did not change for at least 16 h at 15°C. To capture slight changes in proteins, we focused on the amide proton region, which reflects subtle changes around the amino acids that constitute the protein. We initially monitored the structural changes associated with the aggregation of p53C-wt and p53C-R175H without any small additives. A comparison of p53C-wt and p53C-R175H without additives revealed no significant differences in the peak patterns, but sharper peaks were observed for p53C-wt (Figures 4A,D). The peak intensities of both p53C-wt and p53C-R175H decreased markedly over time, probably due to the increase in molecular weight caused by aggregation and the marked decrease in mobility.

With the addition of Glc, there was an increase in peak intensity in both p53C-wt and p53C-R175H, prominent in p53C-R175H (Figures 4B,E). The peaks of p53C-wt with Glc were still detectable after 30 min. However, the peak intensity of p53C-R175H with Glc decreased at the 5-min time point, as with the samples with no additives.

These results of 1D ^1^H-NMR experiments for Glc are consistent with those of the DNA binding assay, which showed that p53C-wt retained its DNA-binding ability in the presence of Glc, whereas p53C-R175H with Glc or samples with no additives lost that ability.

The patterns of the spectra of both p53C-wt and p53C-R175H under 400 mM KCl conditions were different from those under non-additive conditions before aggregation (Figures 4C,F). In the 400 mM KCl condition, a decrease in peak intensity due to aggregation was observed, as in the non-additive condition, but there was no change in the pattern of peaks due to aggregation for both p53C-wt and p53C-R175H. The peaks of p53C-R175H with KCl were still observable after 5 min.

Considering these results together, we propose that the DNA-binding ability of both p53-wt and p53-R175H can be retained by the addition of KCl, and that both the residual peaks and the peak pattern observed in this study might be related to the DNA-binding structure of p53C.

### Detection of Structural Transition by Tryptophan Fluorescence Spectroscopy

**FIGURE 5:**
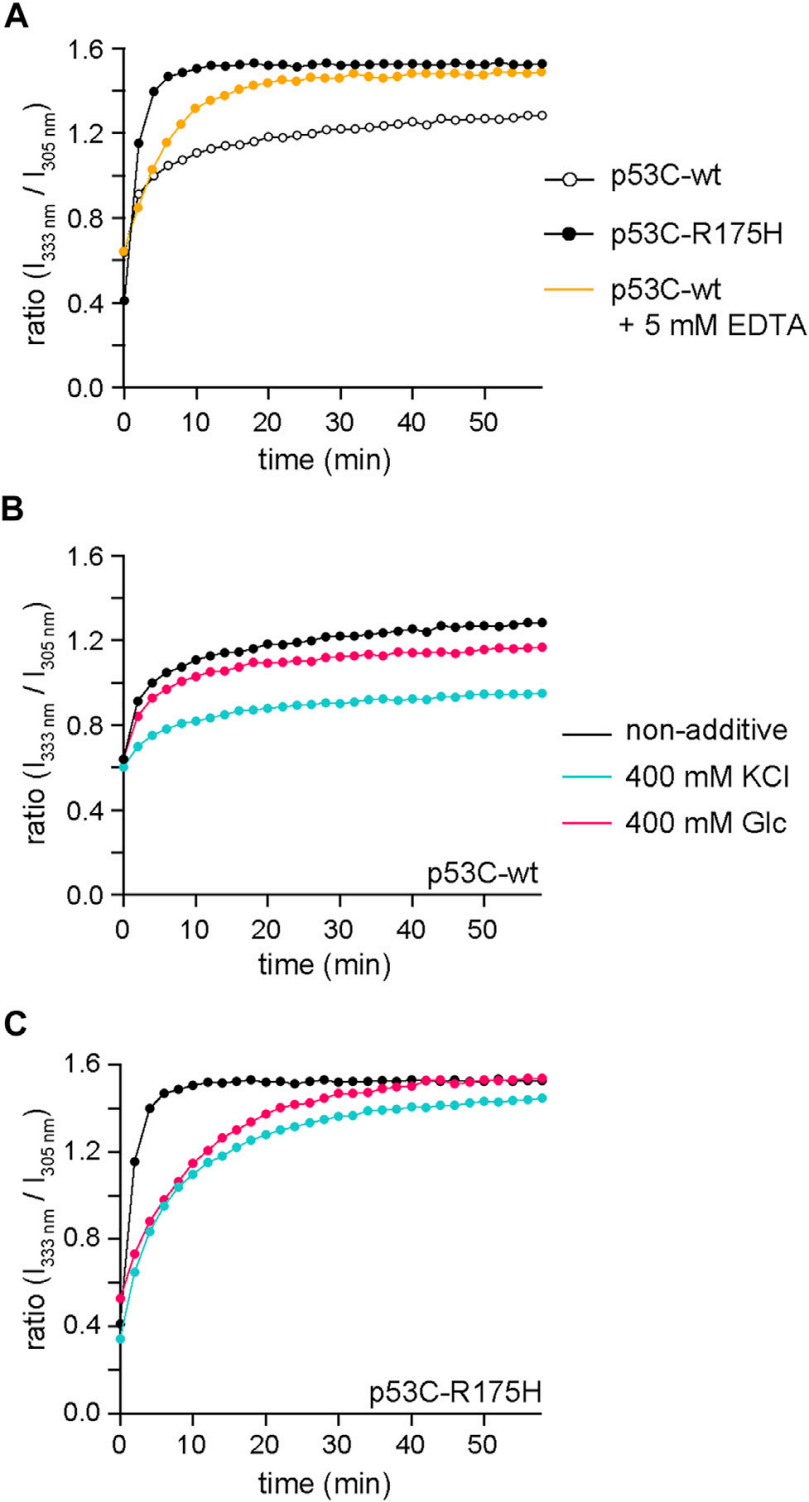
The structural transition was monitored from the ratio of the fluorescence intensity at 333–305 nm when excited at 280 nm. **(A)** Time course of the ratio of the fluorescence intensity at 333 nm to that at 305 nm for p53C-wt without additives (black, open circle), p53C-wt with 5 mM EDTA (yellow), and p53C-R175H without additives (black, solid circle). **(B,C)** Time course of the ratio of the fluorescence intensity at 333 nm to that at 305 nm for p53C-wt **(B)** and p53C-R175H **(C)** without additives (black), with 400 mM KCl (cyan), and with 400 mM Glc (magenta). The data for non-additives are identical to those for Panel **(A)** and have been redrawn for comparison.

The substitution of the 175th arginine with histidine makes it difficult for the Zn ion to coordinate (Butler and Loh, 2003). The structural transition due to Zn removal can be monitored by the change in the ratio of the fluorescence intensity at 305–333 nm when excited at 280 nm. We monitored the structural transition of p53C-wt in the presence and absence of EDTA, as well as that of p53C-R175H without additives (Figure 5A). The profile of p53C-wt with EDTA was close to that of p53C-R175H. The plateau value of p53C-R175H seemed to be the endpoint of the Zn-dislocated structure.

Subsequently, we also monitored p53C-wt and p53C-R175H with 400 mM KCl and 400 mM Glc to reveal the effects of those additives on the structural transition (Figures 5B,C). The results showed that KCl suppressed the structural transition in both p53C-wt and p53C-R175H. Glc also suppressed the structural transition, but the ratio of the final structural transition in p53C-wt was also reduced, whereas in p53C-R175H, the structural transition slowed down, but the ratio of the final structural transition was almost the same as that with no additives. These results are consistent with the observations of NMR experiments, although the protein concentrations were different. This result showed that KCl and Glc could interfere with the structural change due to the loss of Zn.

## Discussion

In this study, we introduced an unconventional experimental strategy to simultaneously monitor p53C aggregation by two different fluorescent probes: ThT and ANS. Some of the results were further assessed using two additional biophysical methods: NTA and NMR. As far as we know, this simultaneous employment of two different fluorescence probes is not popular, probably because its merit is unclear. However, while monitoring p53C aggregation with a single fluorescent probe provides only limited information, the use of multiple fluorescent probes and measuring the excitation spectrum of fluorescence at 485 nm provides various information (Yoshimura et al., 2012; Adachi et al., 2015; So and Yoshimura, 2020). It should be noted that ANS has been reported to increase its fluorescence intensity by binding to the solvent exposed hydrophobic cluster, thereby useful for detecting molten-globules. In this study, we employed ANS to monitor amorphous-like aggregates since it has been reported to be useful to monitor aggregated state (Hawe et al., 2008). We reproduced these previous results by observing a decrease in the fluorescence intensity of ANS upon KCl addition; however, the ThT fluorescence of p53C-R175H increased. Subsequently, we focused on the unique feature of the ratio of the fluorescence of ThT/ANS. Interestingly, although the ratio of p53C-wt or p53C-R175H varied according to the different additives, the ratios were always constant between p53C-wt and p53C-R175H. In other words, only the difference in ions affected the ratio of ThT/ANS, regardless of the mutation on the p53 polypeptide. We assumed that the ratio of ThT/ANS may roughly reflect the ratio of the amount of amyloid-like and amorphous-like aggregates in the system. Thus, according to the basic theorem of chemical reaction kinetics, the ratio is considered to reflect the difference in the relative thermodynamic stability of amyloid-like and amorphous-like aggregates (Thakur et al., 2017). Our observation partially suggested that the amino acid substitution of R175 to H did not affect this difference in the stability of amyloid and amorphous aggregates, whereas R175H only affected the transition states between the folded monomeric p53C (wt and mutant) and each aggregate. Thus, our proposed method, in which simultaneous monitoring of amorphous and amyloid was further analyzed by ThT/ANS fluorescence ratio, is useful for investigating the pathway and the mechanism of p53C aggregation.

We observed both the common and unique effects of the additives KCl and Glc on p53C aggregation. An increase in ThT fluorescence of p53C-R175H was observed upon the addition of KCl and Glc. By contrast, for ANS fluorescence, the opposite responses were observed for KCl and Glc. KCl contributed to reduce amorphous-like aggregate of p53C that were monitored by ANS fluorescence, fluorescent microscopy, and NTA particle analyzer, probably because KCl could suppress the denaturation of p53C. Since ANS binds exposed hydrophobic regions of proteins (Cardamone and Puri, 1992), the increase in ANS fluorescence is attributed to its binding of either the denatured states or amorphous aggregates (Rabbani et al., 2011). Both additives (KCl and Glc) sharpened the ^1^H NMR signals of p53C-wt and p53C-R175H. Taking all these observations into account, KCl and Glc may stabilize the monomeric p53C species, whereas only Glc can stabilize the amorphous aggregate. Thus, the ratio of ThT/ANS fluorescence of p53C-wt and p53C-R175H remained constant with the addition of Glc.

Among the many pathologically relevant hotspot mutants of p53, p53C-R175H, which is classified as a structural mutant, is prone to release the Zn ion, since R175 is spatially close to the coordination site (Butler and Loh, 2003). Zn ions are involved in the DNA-binding activity of p53C (Pavletich et al., 1993). We also reproduced the loss of the DNA-binding ability of p53C-R175H 5 min after aggregation initiation, whereas the DNA-binding ability of p53C-wt remained for 60–90 min. We examined the DNA-binding ability of p53C-wt and p53C-R175H in the presence of KCl and Glc. Surprisingly, the DNA-binding ability of p53C-wt in the presence of KCl and Glc remained at a high level for a long time. Notably, at both conditions, the level of amyloid-like aggregation did not change or even slightly increased, whereas the amorphous aggregates changed in the opposite directions, decreasing (KCl) or increasing (Glc). We concluded that p53C retained its function despite conditions that increased amorphous or amyloid aggregate formation. In other words, an increase in ANS and ThT fluorescence is not directly linked to the loss of the DNA-binding function of p53C. By contrast, the DNA-binding activity of p53C seemed to be related to the proportion of the Zn-coordinating native-like structure monitored by tryptophan fluorescence. We hypothesize that the aggregation process of p53C may contain at least two steps, and that the dissociation of Zn seems prior to either amorphous-like or amyloid-like aggregation.

In the 1D-NMR experiments, p53C showed different spectra in the absence of additives, the presence of 400 mM KCl, and that of Glc. Both KCl and Glc were considered to act on the monomer molecules as stabilizers for the native structure, since many shaper peaks were observed in the presence of these additives. In the case of p53C-R175H, both additives promoted amyloid-like aggregation. Thus, we assumed that p53C forms amyloid aggregates without undergoing a structural transition from its β-sheet-rich monomeric structure, in contrast to other amyloidogenic proteins that undergo a structural transition from their native conformation prior to amyloid formation. Our putative explanation is that stabilizing the Zn-bound form promotes an increase in amyloid-like aggregation. The addition of sugar, especially to p53C-wt, increased the DNA-binding ability, suggesting that the native structure of p53C-wt was stabilized by Glc. However, we observed an increase in ANS fluorescence upon sugar addition, probably because sugar stabilizes the denatured state in the monomeric state and prevents aggregation. The increase in the surface area of p53C may have facilitated ANS binding, resulting in a significant increase in ANS fluorescence. High concentrations of sugars have been shown to inhibit amyloid fibril formation of Aβ (1–42), probably by stabilizing the compact conformation (Iwaya et al., 2020). We assumed that the effect of sugars on p53C was not the case in this study.

Given the structural transition of p53C-wt and p53C-R175H revealed by tryptophan fluorescence, we further assessed the relationship between Zn coordination and the additives. When EDTA was added to p53C-wt to sequester Zn ions, the 333 nm/305 nm fluorescence intensity ratio increased to a plateau value similar to that of p53C-R175H. The fluorescence ratio (333 nm/305 nm) of p53C-wt was suppressed by KCl and Glc, and the ratio (333 nm/305 nm) of p53C-R175H was suppressed by KCl. In all these conditions, the DNA-binding activity of both p53C-wt and p53C-R175H was retained, which suggests that the function of p53-wt is maintained by various small molecules in the cell unlike the *in vitro* system. Note that full-length p53 form liquid-liquid phase separation (LLPS) (Safari et al., 2019) as well as p53C (Petronilho et al., 2021); LLPS is considered to be a precursor to aggregation (Patel et al., 2015; Shin and Brangwynne, 2017). Since aggregation of full-length p53 has been also observed in cancer cells, the findings on p53C in our study may reflect the behavior of full-length p53.

**FIGURE 6:**
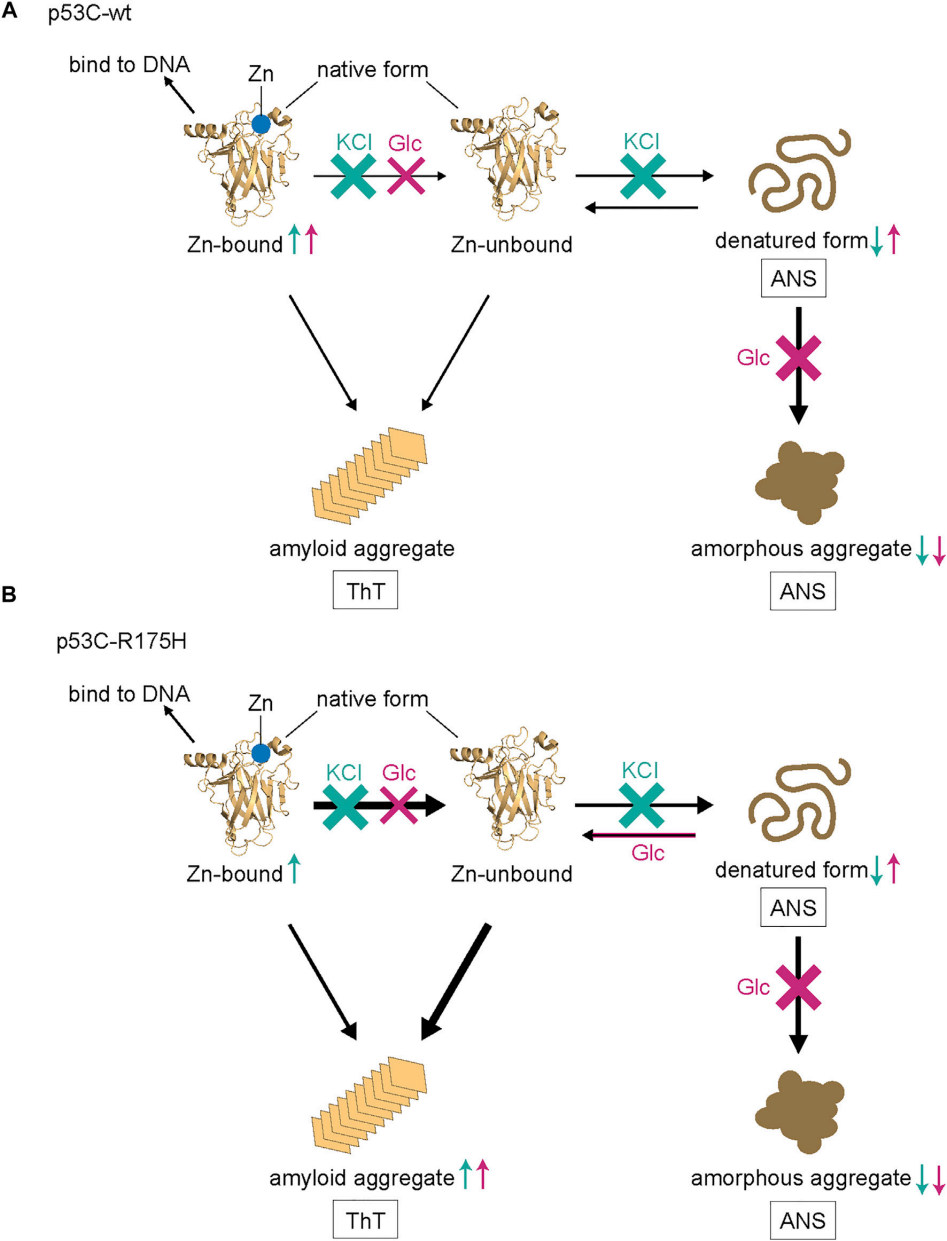
A model of the denaturation and aggregation process of p53C-wt **(A)** and p53C R175H **(B)**. ANS predominantly stains the denatured form and amorphous aggregate, while ThT stains amyloid aggregate. Those that increase or decrease with KCl are represented by cyan arrows, while those that increase or decrease with Glc are represented by magenta arrows, either up or down.

To summarize the molecular mechanisms above, we illustrated a schematic diagram (Figure 6). For p53C-wt, both KCl and Glc showed an increase in the Zn-bound monomer of DNA-binding activity. We assumed that KCl inhibited the two processes—the dissociation of Zn and the denaturation of p53C-wt. For p53C-R175H, KCl increased the DNA binding of the Zn-bound monomer. Although the denaturation was not inhibited by Glc, the increase in monomer molecules suggests that it stabilized the monomer, regardless of whether it was a native or denatured structure. Therefore, we speculate that amyloid aggregates increase due to an increase in the number of molecules that have a native structure but are devoid of Zn. Further investigations are necessary to elaborate on such details of p53C aggregation.

The DNA-binding activity of p53 is indispensable for physiological p53 function as a guardian of the genome that prevents cells from carcinogenesis. We found that the relationship between the DNA-binding activity of p53C and aggregate formation is not straightforward to explain by any single experimental method. Since the structural transition is followed by the formation of amorphous aggregates, the structural transition should become a primary target for preventing p53 aggregation. By contrast, amyloid-like aggregates seem less important in the process of the loss of p53 function, although the prion-like transmission hypothesis of p53 amyloid is still attractive (Ghosh et al., 2017). There are various methods for monitoring p53 aggregation and denaturation, and all the results of each method are often inconsistent. We are also aware that the gel shift assay is not a perfect method, since the additives themselves may affect the DNA-binding process. Nevertheless, it is necessary to analyze p53 aggregation in a combined approach that uses different biochemical and biophysical methods with various small additives.

## Data Availability

The raw data supporting the conclusion of this article will be made available by the authors, without undue reservation.
