# Supplementary material for: Relevance of Amorphous and Amyloid-Like Aggregates of the p53 Core Domain to Loss of its DNA-Binding Activity
Source: Front Mol Biosci. 2022 Apr 26;9:869851. doi: 10.3389/fmolb.2022.869851 (PMC9086241; doi:10.3389/fmolb.2022.869851)
Supplement: Supplementary file 1 [file DataSheet1.PDF]

Supplementary Material

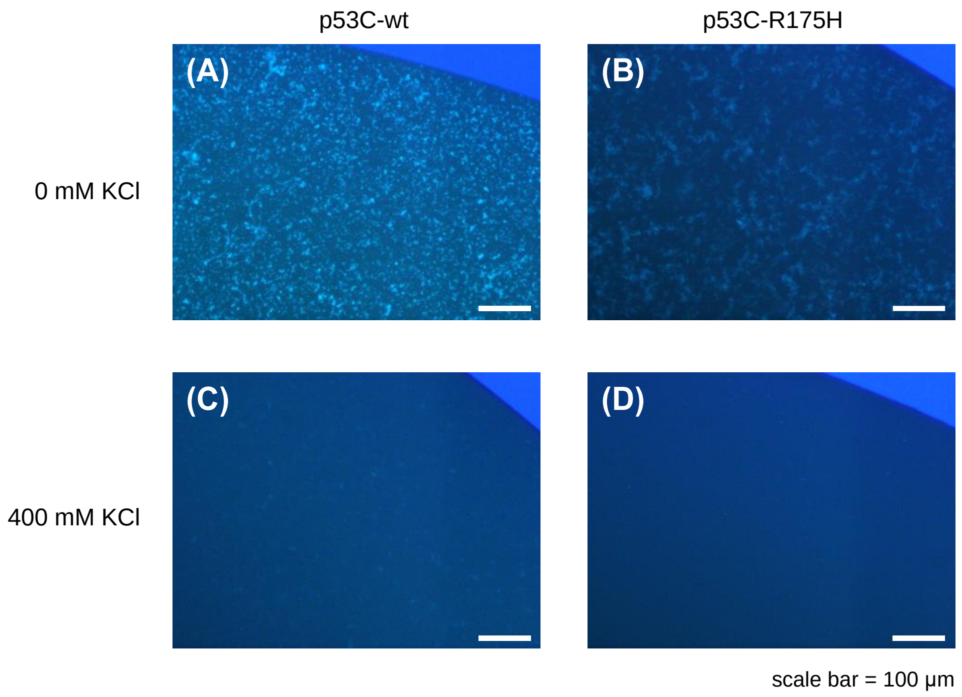

Supplementary Figure 1. Fluorescence microscopy images of wild-type p53C and p53C R175H aggregated at 37°C for 2 hours and stained with ANS.

A, p53C-wt aggregates in the absence of KCl.

B, p53C-R175H aggregates in the absence of KCl.

C, p53C-wt aggregates in the presence of 400 mM KCl.

D, p53C-R175H aggregates in the presence of 400 mM KCl.

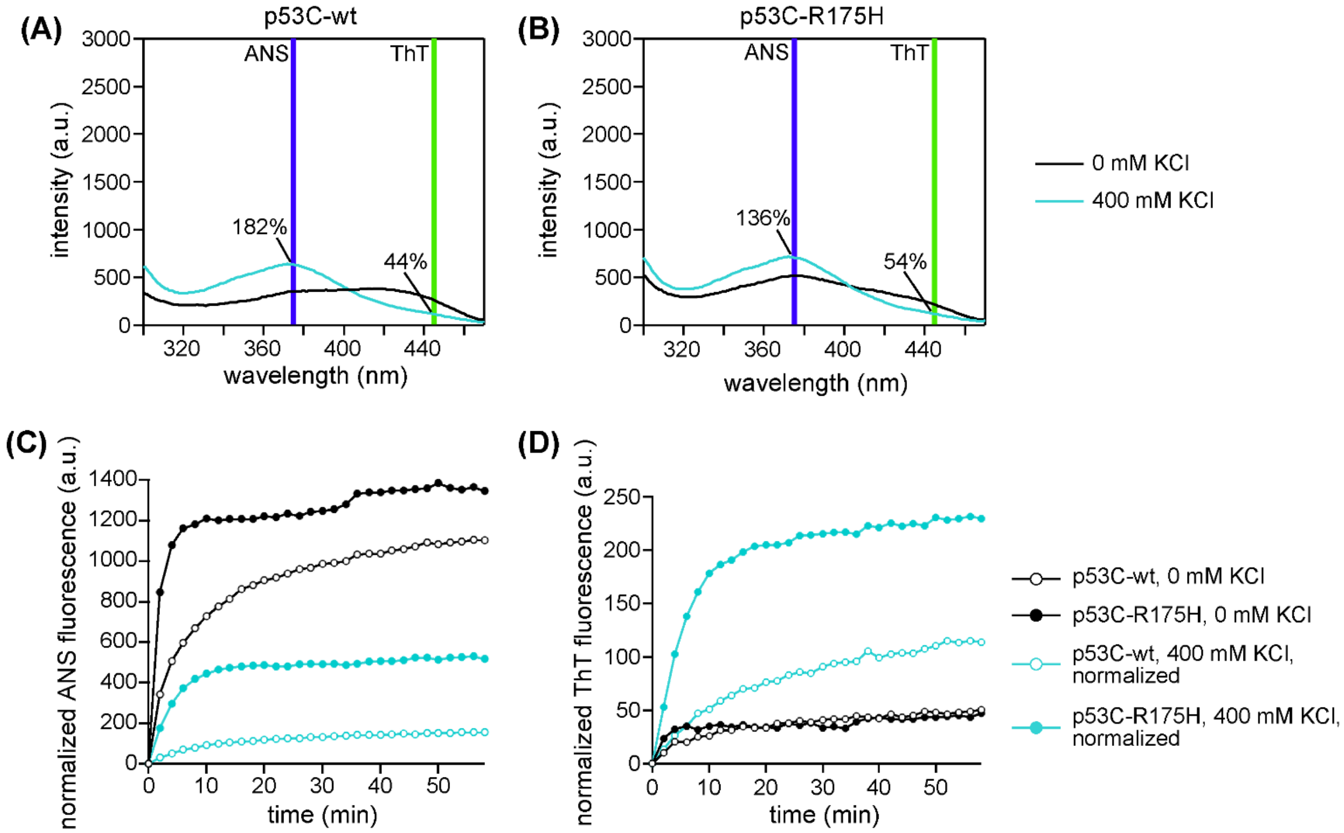

Supplementary Figure 2.

A–B, Effect of KCl to the fluorescence probes against p53C in their native states. The excitation spectra of p53C-wt (**A**) and p53C-R175H (**B**) at fluorescence wavelength of 485 nm. The blue line corresponds to maximal excitation wavelength of ANS fluorescence and the yellow-green line to ThT fluorescence. ANS values changed 182% in p53C-wt and 136% in p53C-R175H in the presence of 400 mM KCl compared to without KCl; ThT values changed 44% in p53C-wt and 54% in p53C-R175H in the presence of 400 mM KCl compared to without KCl.

C–D, ANS and ThT fluorescence in the presence of 400 mM KCl was normalized by taking into account the fluctuations due to 400 mM KCl shown in panel **A** and **B**.

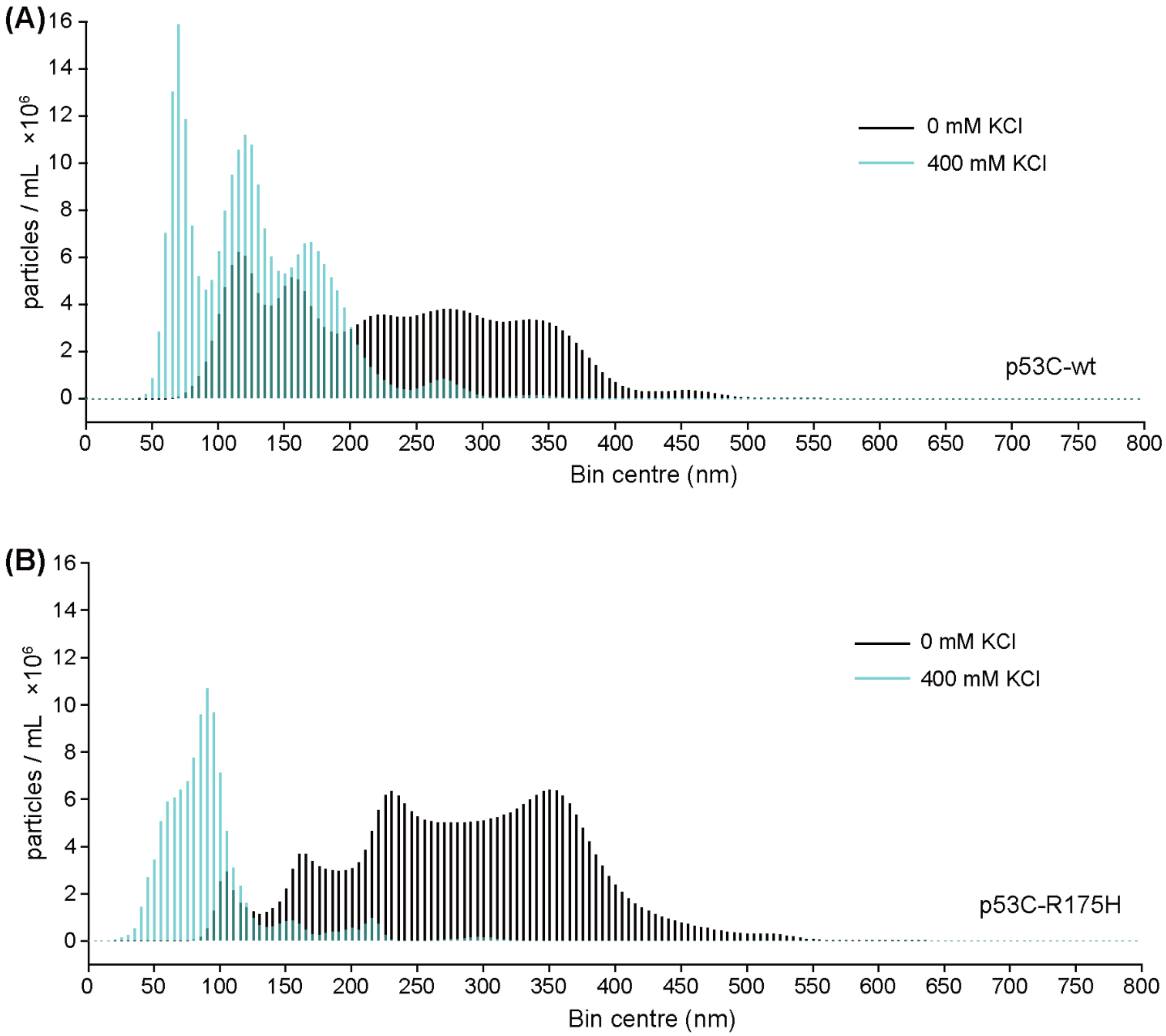

Supplementary Figure 3. Histogram representation of particle numbers analyzed by NTA. The size of the aggregates of p53C-wt (**A**) and p53C-R175H (**B**) formed in the absence of KCl (black bars) or in the presence of 400 mM KCl (cyan bars) was divided into 5 nm increments. The data are identical to those in Fig. 1F.

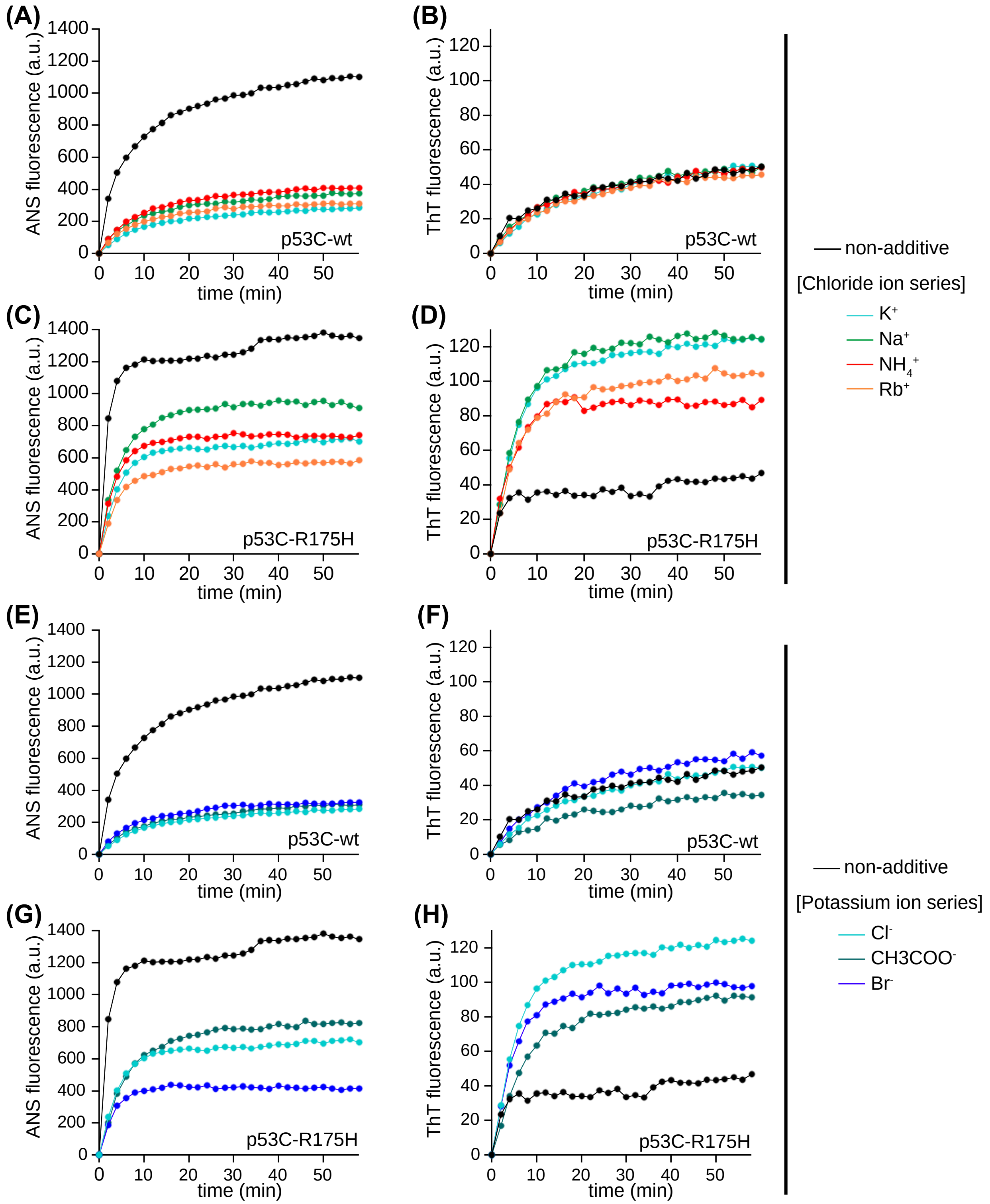
(A)
ANS fluorescence (a.u.)
time (min)
p53C-wt
(B)
ThT fluorescence (a.u.)
time (min)
p53C-wt
(C)
ANS fluorescence (a.u.)
time (min)
p53C-R175H
(D)
ThT fluorescence (a.u.)
time (min)
p53C-R175H
(E)
ANS fluorescence (a.u.)
time (min)
p53C-wt
(F)
ThT fluorescence (a.u.)
time (min)
p53C-wt
(G)
ANS fluorescence (a.u.)
time (min)
p53C-R175H
(H)
ThT fluorescence (a.u.)
time (min)
p53C-R175H
non-additive
[Chloride ion series]
K+
Na+
NH4+
Rb+
non-additive
[Potassium ion series]
Cl-
CH3COO-
Br-

Supplementary Figure 4. Effect of various ions on aggregation of p53C-wt and p53C-R175H.

A–D, Chloride salts of K^+ (cyan), Na^+ (green), NH_4^+ (red), and Rb^+ (orange)were added. **A,** ANS fluorescence in p53C-wt; **B,** ThT fluorescence in p53C-wt; **C,** ANS fluorescence in p53C-R175H; **D,** ThT fluorescence in p53C-R175H.

E–H, Potassium salts of Cl^- (cyan), CH_3COO^- (dark green), and Br^- (blue) were added. **E,** ANS fluorescence in p53C-wt; **F,** ThT fluorescence in p53C-wt; **G,** ANS fluorescence in p53C-R175H; **H,** ThT fluorescence in p53C-R175H.

The data for non-additives are identical to those in **Fig. 1A–D**, and have been redrawn for comparison.

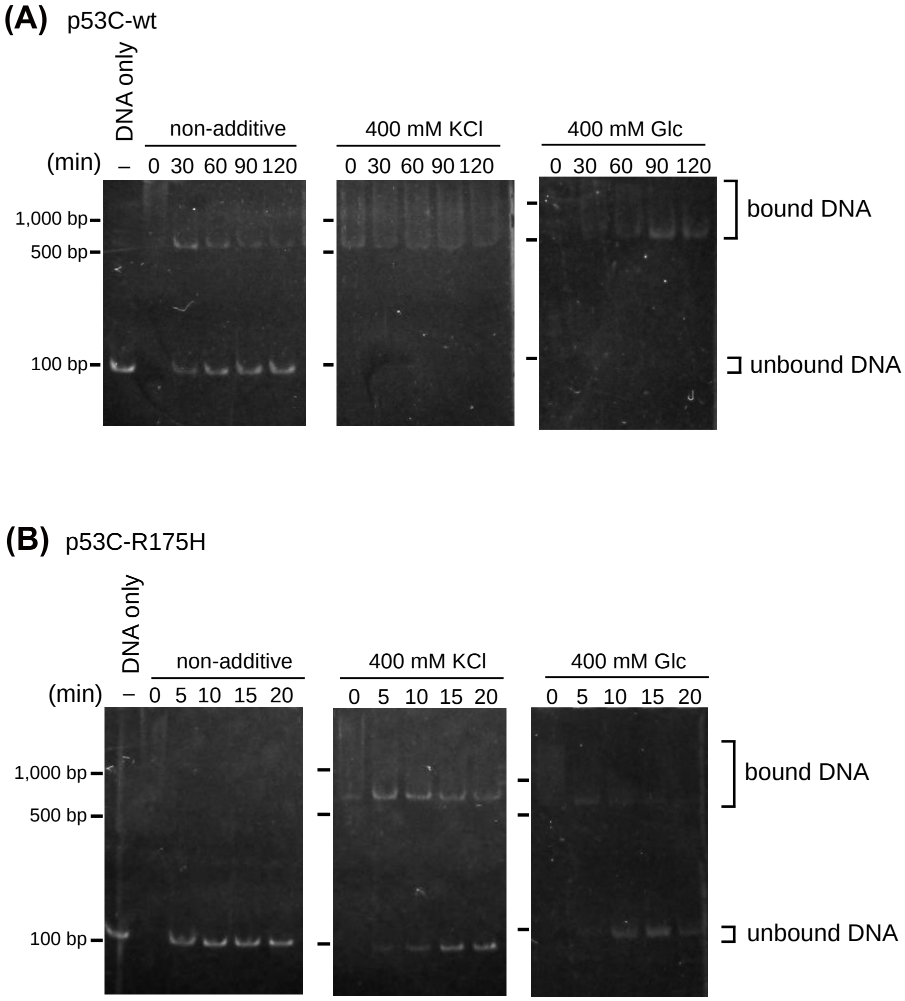

Supplementary Figure 5. Gel shift assay with p53C solution and DNA containing the p53 binding sequence. The electrophoresed gel was stained with ethidium bromide to detect DNA.

A, In the absence of additives, in the presence of 400 mM KCl, or in the presence of 400 mM Glc, p53C-wt was aggregated for 0, 30, 60, 90, or 120 min and then bound to DNA.

B, In the absence of additives, in the presence of 400 mM KCl, or in the presence of 400 mM Glc, p53C-R175H was aggregated for 0, 5, 10, 15, or 20 min and then bound to DNA.
